# Genetic variation of transgenerational plasticity of offspring germination in response to salinity stress and the seed transcriptome of *Medicago truncatula*

**DOI:** 10.1186/s12862-015-0322-4

**Published:** 2015-04-01

**Authors:** Wendy T Vu, Peter L Chang, Ken S Moriuchi, Maren L Friesen

**Affiliations:** Section of Molecular and Computational Biology, Department of Biology, University of Southern California, Los Angeles, USA; Plant Pathology, University of California at Davis, 116 Robbins Hall, Davis, CA USA; Department of Plant Biology, Michigan State University, East Lansing, MI USA

**Keywords:** Transgenerational plasticity, Parental environmental effects, Stored seed transcripts, Germination, Genetic variation

## Abstract

**Background:**

Transgenerational plasticity provides phenotypic variation that contributes to adaptation. For plants, the timing of seed germination is critical for offspring survival in stressful environments, as germination timing can alter the environmental conditions a seedling experiences. Stored seed transcripts are important determinants of seed germination, but have not previously been linked with transgenerational plasticity of germination behavior. In this study we used RNAseq and growth chamber experiments of the model legume *M. trucantula* to test whether parental exposure to salinity stress influences the expression of stored seed transcripts and early offspring traits and test for genetic variation.

**Results:**

We detected genotype-dependent parental environmental effects (transgenerational plasticity) on the expression levels of stored seed transcripts, seed size, and germination behavior of four *M. truncatula* genotypes. More than 50% of the transcripts detected in the mature, ungerminated seed transcriptome were annotated as regulating seed germination, some of which are involved in abiotic stress response and post-embryonic development. Some genotypes showed increased seed size in response to parental exposure to salinity stress, but no parental environmental influence on germination timing. In contrast, other genotypes showed no seed size differences across contrasting parental conditions but displayed transgenerational plasticity for germimation timing, with significantly delayed germination in saline conditions when parental plants were exposed to salinity. In genotypes that show significant transgenerational plastic germination response, we found significant coexpression networks derived from salt responsive transcripts involved in post-transcriptional regulation of the germination pathway. Consistent with the delayed germination response to saline conditions in these genotypes, we found genes associated with dormancy and up-regulation of abscisic acid (ABA).

**Conclusions:**

Our results demonstrate genetic variation in transgenerational plasticity within *M. truncatula* and show that parental exposure to salinity stress influences the expression of stored seed transcripts, seed weight, and germination behavior. Furthermore, we show that the parental environment influences gene expression to modulate biological pathways that are likely responsible for offspring germination responses to salinity stress.

**Electronic supplementary material:**

The online version of this article (doi:10.1186/s12862-015-0322-4) contains supplementary material, which is available to authorized users.

## Background

Transgenerational plasticity occurs when the parental environment influences offspring development and responses to environmental conditions in the absence of genetic changes. While transgenerational plasticity is not necessarily adaptive, it is predicted to influence the rate of adaptation by changing the strength and direction of responses to selection in the offspring generation [1,2]. Evolutionary theory shows that transgenerational plasticity can sometimes be an adaptive mechanism that increases long-term fitness under environmental heterogeneity [3-5] and extreme environmental shifts [2,6]. Parental exposure to predation in three-spine sticklebacks and crickets have been shown to adaptively influence offspring anti-predator behavior [1,2,7,8] and defense phenotypes of wild radish progeny have been correlated with parental exposure to herbivory [3-5,9]. However, in situations where environments are highly unpredictable [10,11] or conditions fall outside the adaptive range of a population [12,13], transgenerational plasticity can also be maladaptive. In order for transgenerational plasticity to itself evolve, it must be heritable and exhibit variation between genotypes. There is evidence that transgenerational plasticity is genetically based in field studies examining the influence of the parental environment on offspring response and performance relative to contrasting parental environments [2,6,12,14]. Several studies in plants have reported genotypic differences in adaptive transgenerational plasticity [12,15,16], where genotypes differ in the type of transgenerational mechanisms responsible for the transmission of adaptive environmental cues to their offspring.

Plants are an attractive model system for transgenerational plasticity because of the ease of manipulating parental and offspring environments. Plants exhibit high levels of phenotypic plasticity since they are sedentary organisms with little choice in their growth environment [17,18]. Because seed dispersal is often limited to the range of the parental plant’s environment [19], the parental environment is likely a good predictor of the offspring environment, which would favor transgenerational environmental cues [2,6,20-22]. Experimental manipulations showed that transgenerational plasticity in *Campanulastrum americanum* is adaptive in the wild, but only when the parental environment is a predictor of the offspring environment [12].

Plants have two well-characterized processes that mediate transgenerational plasticity in offspring performance and response: resource provisioning as reflected in seed size [23,24] and seed dormancy/germination pathways [25,26]. Seed size is often linked directly to performance variation through growth rate and competitive ability [27,28], while the dormancy pathways determine germination timing—an important life history trait that determines the environment experienced by the developing plant [29]. In addition to dormancy pathways, seed size is often a determinant of germination behavior in some but not all species [30-32]. In *Pinus pinaster*, parental environmental effects on seed mass partially explained variation in germination timing between contrasting parental environments [33].

Dormancy is an adaptive life history trait to seasonally unfavorable environmental conditions [29,34]. Dormancy is established during seed development on the parental plant [35]; therefore, this trait is likely programmed during seed development. There are a variety of mechanisms that have evolved to mediate seed dormancy in angiosperms. Physical seed dormancy is mediated by the seed coat (“hardseededness” or “seed hardiness”) [36-38]. The seed coat is a maternal tissue that is made of a waxy, hydrophobic tegument that prevents the uptake of water and oxygen required for germination [39,40] and is likely under the control of the maternal genotype [23,41]. Another mechanism is physiological dormancy, which is represented by two classes: primary and secondary dormancy. Primary dormancy is maintained by the accumulation of phytohormone abscisic acid (ABA) during seed maturation to prevent precocious seed germination [38], and requires a period of after-ripening before seeds have the capacity to germinate under favorable conditions. Secondary dormancy, on the other hand, is the re-induction of dormancy by after-ripened non-dormant seeds in response to certain environmental conditions [36,42-44], particularly unfavorable ones [23,41]. Imbibed after-ripened seeds of *Arabidopsis thaliana* can induce secondary dormancy under certain temperature regimes through changes in the expression of dormancy related transcripts [45,46], suggesting that transcriptional regulation can mediate delayed germination response to offspring environmental condition.

Transcripts stored in mature seeds play a critical role in regulating seed germination and dormancy in a variety of plant species [47-52]. Work in *A. thaliana* using chemical inhibitors of transcription and translation demonstrated that stored seed transcripts are both necessary and sufficient for seed germination [53]; variation in stored seed transcript expression of VIVIPAROUS was related to the length of time required for seeds to break dormancy in *Avena fetua* [49]. Stored seed transcripts, like other compounds important for early seedling establishment, are deposited after embryogenesis and during seed maturation [54,55] and previous studies have found that expression profiles of stored seed transcripts respond to the parental environment [35,50].

*Medicago truncatula*, a member of the Fabaceae family, is primarily a selfing annual legume native to the Mediterranean region and found naturally occurring in both saline and non-saline habitats [56-59]. In saline habitats, salt accumulates at the surface soil during the summer, with soil salt concentrations peaking during the first rain from the summer drought and then dropping as additional rains leach salt from the soil surface [60,61]. One mechanism by which pasture legumes adapt to saline environments is by delaying germination to avoid high salt concentrations early in the rainy season [62]. Along with other *Medicago* species, *M. truncatula* is characterized to exhibit both seed coat-imposed physical dormancy and physiological primary dormancy [36,39,63-65], with ABA playing an important role in the latter [63]. Although *M. truncatula* exhibits non-deep primary dormancy (several weeks/months of after-ripening is sufficient to remove dormancy), it is unclear whether secondary dormancy occurs in *M. truncatula* seeds [38,63]. While the *M. truncatula* transcriptome of developing and dry, mature seeds has been previously characterized [66], it is currently unknown whether the parental environment influences stored seed transcripts and whether these transcripts play a role in seed dormancy or delayed germination responses.

In this study, we explore the molecular mechanisms that are influenced by the parental environment to facilitate offspring transgenerational responses. We use wild genotypes of the model legume *M. truncatula* from naturally saline and non-saline habitats to explore parental environmental effects on the expression of stored seed transcriptome, which could potentially mediate transgenerational plasticity in response to salinity stress. We focus on seed dormancy and identify genes and pathways potentially responsible for transgenerational plasticity of germination behavior. We test the hypothesis that *M. truncatula* shows genetic variation for transgenerational plasticity of germination timing under parental salinity stress. Using next-generation sequencing, we assess the influence of parental salt exposure on the stored seed transcriptome and determine whether parental environmental effects on seed transcript expression differ among genotypes. Finally, we identify novel candidate biological pathways influenced by the parental environment to facilitate offspring transgenerational plastic germination behavior*.*

## Methods

Self-fertilized seeds derived from four inbred Tunisian genotypes of *M. truncatula*, TN1.13, TN1.15, TN7.22, and TN8.22, were used to measure transgenerational plasticity (parental environmental effects) in early developmental phenotypes. These genotypes are a subset of a larger collection of 39 genotypes derived from two saline (i.e., Enfidha:TN1 and Soliman:TN8) and two non-saline (i.e., El Kef:TN7 and Bulla Regia:TN9) populations [56,58,59]. TN1.13 and TN1.15 originate from the same saline source population (TN1), TN8.22 is from the TN8 saline population (TN8), and TN7.22 is from the TN7 non-saline population [57]. These genotypes were chosen from a population scale parental environmental effects experiment of all 39 Tunisian genotypes in UC Davis [67]. TN1.13 and TN1.15 were chosen because they displayed significant transgenerational plasticity, while TN8.22 and TN7.22 were selected because they exhibited no patterns of parental environmental influence on offspring germination behavior.

### Parental and offspring environment

During the parental generation, seeds from each genotype were grown in a 2:1 sterile horticultural sand: UC Davis soil mix in 4 cm diameter/20 cm long cone-tainers (Steuwe) under ambient conditions at UC Davis. Two weeks after germination, salt treatments were initiated by treating half of the plants with a Fahraeus solution with either 0 mM NaCl or 100 mM NaCl (i.e., parental environment). 100 mM NaCl is within the range of salinity observed in the field, which is sufficient to cause stress but not extreme mortality. For the offspring generation, ten seeds from each genotype and parental environment were planted in growth chambers at USC and the conditions were set at 16/8 hour day/night cycle with temperatures at 13°C and 18°C (similar to the growth conditions experienced by the parental plants). Seeds were after-ripened for one year in dry conditions before they were used for the offspring germination experiment. Single, unvernalized seeds were weighed and sandpaper scarified and planted into the same growth medium as the parental plants. Pots were fully randomized every two weeks until the end of the experiment and seeds were immediately treated with Fahraeus nutrient solution spiked with either 0 mM or 100 mM NaCl and subsequent treatments were done twice a week. We recorded the timing of germination, unifoliate development, and first trifoliate development. Plants were surveyed daily and seeds were considered germinated when cotyledons were fully expanded. All plants were grown to senescence and pods were collected as they naturally matured on each plant.

### Transcriptome library construction

To test the effects of the parental environment on seed transcripts, we constructed Illumina Solexa sequencing libraries of 24 self-fertilized dry, ungerminated seeds derived from the same lot of seeds used for the phenotyping. The seeds experienced 1 year of after-ripening before they were used for this experiment. For each genotype and parental environment, we had three biological replicates that consist of a single seed per replicate. For each library, mRNA was isolated from a single seed using Dynabeads mRNA DIRECT Kit from Invitrogen (Product # 610.2, Grand Island, NY) and fragmented using Ambion mRNA Fragmentation Kit (Product #AM8740, Grand Island, NY), followed by cDNA synthesis using random hexamer primers. Double stranded cDNA fragments were blunt-end repaired using Epicentre End Repair (Product #ER81050, Madison, WI) and added a single A to the blunt end using Klenow Fragment 3′-5′ exo-nuclease (NEB Product #M0212L, Ipswich, MA). Ilumina adaptors were ligated to the cDNA fragments using the Epicentre Fast-Link DNA Ligation Kit (Product #LK6201H, Madison, WI). Fragments between 200-400 bp were size selected by agarose gel and samples were indexed and enriched. The libraries were indexed using 12 indices and sequenced on two lanes using the Illumina GAIIx, which generated 76 bp single-end reads.

### Mapping and normalization of sequencing reads

To identify which transcripts are stored in seed tissues across the experiment, all reads from all samples were pooled and mapped to the *M. truncatula* 3.5.1 genome sequence. We used Tophat to map reads to the genome and generated full-length fragments [68]. These fragments were assembled using Cufflinks [68] to identify the corresponding gene annotations. To identify differential expression patterns, the sequenced reads from each sample were then analyzed independently using Cufflinks to generate counts and coverage for seed-expressed genes and their isoforms. All samples were normalized using the TMM protocol implemented using the edgeR package in R [69], which takes into account differences in overall RNA populations across biological samples.

### Offspring phenotype data analysis

The influence of genotype (G), parental environment (PE), and offspring environment (OE) on offspring traits (i.e. germination timing, timing of unifoliate and first trifoliate development, leaf size, leaf number) was analyzed using the ANOVA package in R. We considered all possible interactions between the main effects, and included seed weight as a covariate. Data were Natural logarithm and square root transformation prior to analyses to satisfy the model’s assumptions of normality and homoscedasticity. Genetic variation in transgenerational plasticity would be indicated by a significant GxPE or GxPExOE interaction with Bonferroni correction.

### Seed transcriptome data analysis

For the seed transcriptome data, we used a negative binomial generalized linear model to analyze the contributions of G, PE, and their interaction on the expression level of 4,358 expressed genes. Within a genotype-treatment combination, a gene was classified as expressed when at least two out of three biological replicates had FPKM values greater than 1. We analyzed the contributions of the G and PE on the level of gene expression only for genes that were expressed in all four genotypes. We used MASS (http://stat.ethz.ch/R-manual/R-patched/library/MASS/html/glm.nb.html), to run the negative binomial generalized linear model and analyzed the contribution of PE for the individual genotypes using a false-discovery rate threshold of FDR < 0.05. The seed transcriptome was analyzed for overrepresentation of biological processes terms using GOstat [30] and GO annotations were obtained from the Noble Foundation (http://mtgea.noble.org/v3/). The data set supporting the results of this article is available in the NCBI Short Read Archive repository under ID SRP012122.

### Network and functional analysis

Seed coexpression network topology file was downloaded from the SeedNet database (bree.cs.nott.ac.uk/arabidopsis/) and visualized using Cytoscape version 2.8.3 [70]. Cytoscape was used for network visualization and functional analysis of the seed transcriptome [70]. The jActiveModules plugin was used to identify the interaction modules referenced from a model of genome-wide transcriptional interaction network derived from publically available microarray expression data of *Arabidopsis* mature seeds from the SeedNet database [51]. Putative sub-networks with aggregate Z-scores greater than 3.0 are generally considered significant and sub-networks were chosen for analysis according to highest ranked Z-scores. Because jActiveModules relies on random sampling, we ran several iterations of the data set to ensure the reproducibility of the identified modules. To extract meaningful molecular associations from the complex sub-networks identified, transcriptional interaction modules were further partitioned into tightly linked coexpression clusters using the MCODE plugin [71]. MCODE cluster scores greater than 2.0 were considered meaningful [72] and clusters analyzed for this study were selected by highest ranked. Since we assume that genes in a module are involved in the same biological process, the predicted modules and clusters were validated by determining if the interacting nodes (genes) are enriched for any Gene Ontology (GO) biological processes using the BiNGO plugin. The hypergeometric test along with the Benjamini and Hochberg false discovery rate correction for multiple testing with a p-value threshold of 0.001 were used to identify significant overrepresented GO terms [73].

## Results

Stored seed transcripts play a critical role in germination [53] and could potentially mediate transgenerational plastic germination responses; therefore, we sequenced the transcriptome of dry, mature seeds originating from parental plants exposed to saline and non-saline conditions. A subset of the mature dry seeds was used for transcriptome sequencing of stored seed transcripts, while the other subset of seeds were used to quantify seed size and germination timing in saline and non-saline offspring conditions. Because *M. truncatula* seeds are self-fertilized, the parental environmental effect incorporates both maternal and paternal effects. We note that these seeds were dry and hence the transcript accumulation is not influenced by seed germination but rather reflects the deposition of storage transcripts during seed maturation.

### Sequencing stored seed transcriptome

We report an average of 1.5 million mapped 76 bp reads for each library, resulting in ~6X coverage of sequenced genes. Though our results are conservative, we identified 9,281 genes expressed in seeds, which is more than the 2,759 genes identified in *M. truncatula* mature seeds [66] and less than the ~12,000 genes expressed in *Arabidopsis* seeds [74] and the 17,000 genes expressed in rice [75]. Gene ontology (GO) enrichment analysis revealed significant enrichment of biological processes (Additional file 1), some of which are involved in gene expression (GO:0010467), RNA metabolic processes/RNA processing (GO:0016070, GO:0006396), translation (GO:0006412), response to osmotic stress (GO:0006970), chromatin modification (GO:0016568), RNA splicing (GO:0008380) and miRNA-mediated gene silencing (GO:0035196). Furthermore, *M. truncatula* forms symbiotic relationships with nitrogen-fixing rhizobia and we find significantly under-enrichment of genes involved in nodulation (GO:0009877) and symbiosis (GO:0044419) processes in the seed transcriptome, suggesting that the symbiosis pathway is independent of the germination pathway.

### Stored seed transcripts are annotated to be involved in germination and dormancy processes

We then asked whether the transcripts detected in *M. truncatula* overlapped with those known to be involved in germination in *A. thaliana.* Using the gene annotations of *A. thaliana* homologues, we compared the genes expressed in our seed transcriptome with genes characterized in the *Arabidopsis* seed coexpression network [51]. We found that 58% of genes expressed in *M. truncatula* seeds (5359/9281) are involved in regulating seed germination and dormancy processes, accounting for 62% of genes represented in the *Arabidopsis* seed network (5359/8621). This overlap is disproportionately higher than expected by chance (Fisher-test, p-value = 0.0025), indicating that we captured seed transcripts that are involved in regulating germination and dormancy processes. Among the genes found in the seed network, we find significant enrichment for biological pathways involved in abiotic stress response (GO:0009628), cellular process (GO:0009987), cellular nitrogen compound metabolic process (GO:0034641), post-embryonic development (GO:0009791) and protein transport (GO:0015031).

### Genotype-dependent parental environmental effects on stored seed transcripts

To examine the genetic basis of transgenerational plastic responses to salinity stress, we exposed four parental genotypes to saline and non-saline environments and collected seeds from each genotype per parental environment. To quantify the genetic differences in the expression of the transcriptome of dry, mature seeds, we tested genotype (G) and parental environmental effects (PE) on the expression of stored seed transcripts. A GxPE interaction indicates genotypic differences in parental environmental influence on the expression of stored seed transcripts. Correcting for multiple testing, we found 1,362 genes that respond to the parental environment in a genotype dependent way (GxPE; all FDR < 0.05), along with 1,500 genes that vary in expression across genotypes (G) irrespective of the parental environment, and 471 genes that are responsive to the parental environment (PE) across all genotypes.

Because we detected genetic variation in parental environmental effects (GxPE) on the expression levels of stored seed transcripts, we analyzed TN1.13, TN1.15, TN7.22 and TN8.22 separately, and detected 1195, 327, 125 and 82 genes differentially expressed between parental environments, respectively. Furthermore, there is minimal overlap of PE responsive transcripts between the genotypes and no genes shared among all four genotypes, suggesting genotypic differences in salt stress response mechanisms. Genotypes TN1.13 and TN1.15 possessed significantly more PE responsive transcripts than genotypes TN7.22 and TN8.22 (Fisher Test, p-value = 3.227e-08).

### Genotype-dependent transgenerational plasticity on germination behavior

To quantify the genetic basis of transgenerational plastic germination behavior, we tested G, PE, OE and all possible interactions on germination timing. A significant OE term indicates germination plasticity in response to the offspring environment, while a significant PE term indicates parental environmental influence on offspring’s germination response. A significant PExOE indicate transgenerational plasticity or parental environmental effect that is dependent on offspring environment. A GxPExOE interaction indicates genetic variation of trangenerational plasticity on offspring germination response that is dependent on the relationship of the parental and offspring environment. Given that we detected genotypic effects in the expression levels of stored seed transcripts and that previous experiments with these genotypes found phenotypic differences in transgenerational plasticity [67], we predict genetic variation in transgenerational plasticity of germination timing. Indeed, we find genotype-dependent transgenerational plasticity (GxPE) on germination timing of the same seed lot in which we measured stored seed transcript differences.

**Figure 1:**
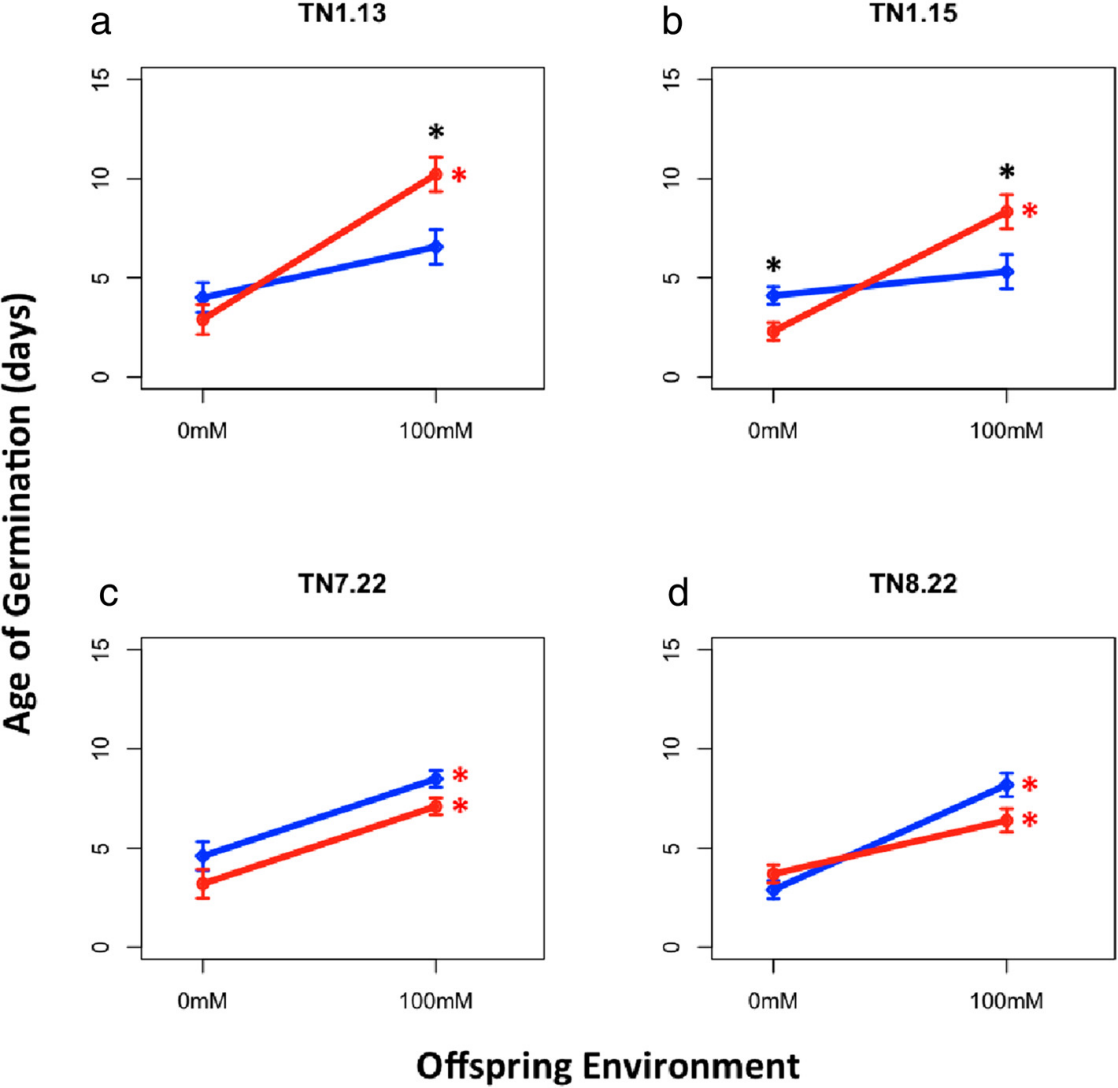
**Norms of reaction plots for germination timing in response to parental and offspring environment.** Blue and red lines correspond to 0 mM and 100 mM NaCl parental environment, respectively. The black asterisk indicates significantly different means with respect to parental environments, and red asterisk indicates significantly different means with respect to offspring environment (Mann–Whitney Test, *P <* 0.05).

We detected a significant three-way interaction (GxPExOE) for germination timing that reflects genotype dependent transgenerational plasticity on germination behavior, timing of unifoliate and first trifoliate development (F_(3,138)_ = 7.75, *P* < 0.00001; Additional file 2). Consistent with the expectations from preliminary germination data, TN1.13 and TN1.15 offspring were the only genotypes that exhibited germination timing that depended on the interaction between the parental and offspring environment (PExOE, F_(1,32)_ = 9.366, F_(1,34)_ = 19.964; Bonferroni correction: *P* < 0.00125; Additional file 3). These genotypes only show significant differences in germination responses to the offspring environment when the parental environment was saline, but no such differences were seen when the parental environment was non-saline (Figure 1a and b, red asterisk). In contrast, TN7.22 and TN8.22 displayed no significant transgenerational plasticity on germination timing (TN7.22: F_(1,34)_ = 0.297, *P* > 0.0125; TN8.22: F_(1,35)_ = 3.107, *P* > 0.0125; Figure 1c and d, Additional file 3), but germination differences in response to salinity were primarily driven by the offspring environment (Figure 1c and d, Additional file 3).

In summary, these results show genotype-dependent transgenerational plasticity of offspring germination timing. Among the genotypes that express transgenerational plastic germination response (TN1.13 and TN1.15), offspring plasticity was dependent on the parental environment: only parental plants that experienced saline conditions produced offspring with plastic germination responses to offspring environment (Figure 1). Furthermore, we find that the offspring environment has a larger effect on offspring germination timing than transgenerational effects (Additional file 3).

### Genotype-dependent parental environmental effects on seed size and the absence of seed size effects on germination behavior

**Figure 2:**
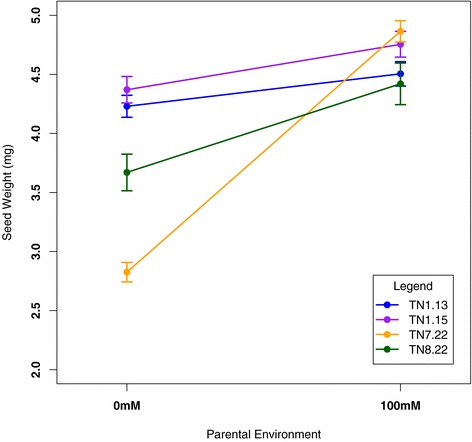
**Seed weight comparison of genotypes developing in 0 mM and 100 mM parental NaCl conditions.**

We test both genetic and parental environmental effects on seed size variation and examine the relationship of seed size on germination behavior. Seed size differences between saline and non-saline parental conditions varied among genotypes: the parental environment significantly influenced seed size in genotypes TN7.22 and TN8.22, but had no influence on seed size in genotype TN1.13 and TN1.15 (Figure 2). TN7.22 and TN8.22 parental plants exposed to saline conditions produced larger seeds relative to non-saline conditions, while TN1.13 and TN1.15 parental plants produced seeds that did not differ in size relative to treatment conditions (Figure 2). Because parental environmental effects on seed size is often correlated with germination timing [26,30,31,33], we tested the effects of seed size on germination timing by including it as a covariate in the analysis of variance. We found no relationship between seed mass and germination timing across all four genotypes (Additional files 2 and 3).

### Salt responsive mature seed transcripts are involved in dormancy and ABA-related processes

Since an organism’s response to environmental cues are not guided by the expression changes of just one gene but rather a network of interacting genes, finding differentially expressed transcripts may not tell us much about how these transcripts work together to influence the expression of a trait. Coexpression networks devised from correlated gene expression is a powerful approach to find functional relationships between changes in gene expression and phenotypic response. Coexpression networks work on a basic principle that genes involved in a biological pathway are co-regulated; thus, coexpressed genes are more likely to function in the same biochemical or developmental pathways [76-80].

**Figure 3:**
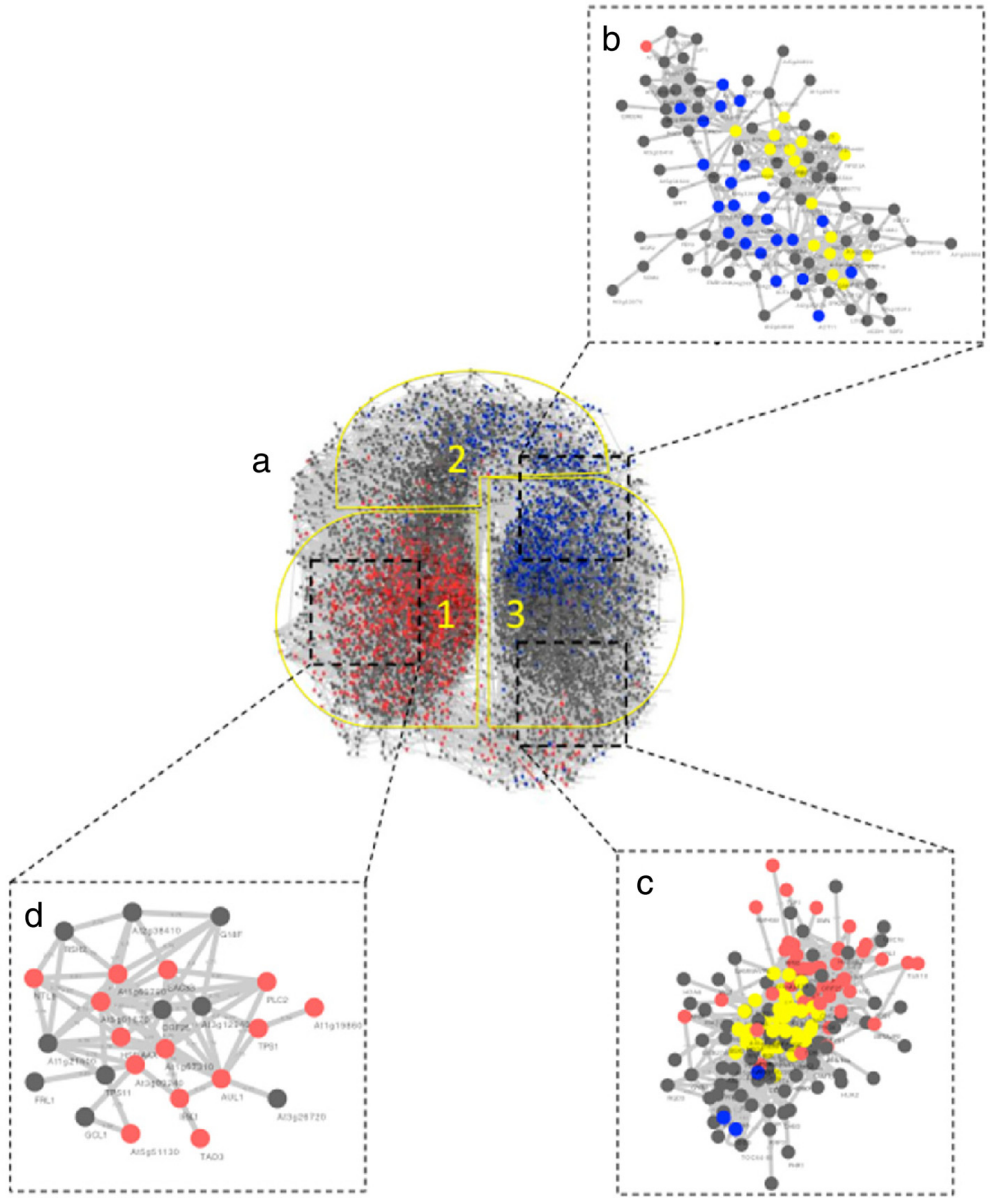
**Seed coexpression network topology. (a)** The *Arabidopsis* germination coexpression network. Outlined in yellow are defined regions in the network: region 1 represents clusters of genes associated with nongermination/dormancy; region 2 represents a transition from nongerminating/dormancy to germinating states; region 3 is associated with germination. Subnetworks generated from salt responsive transripts expressed in genotypes **(b)** TN1.13 and **(c)** TN1.15. **(d)** Subnetwork of overlapping salt responsive genes of TN1.13 and TN1.15. The red and blue nodes represent transcripts significantly associated with the upregulation of dormancy and germination, respectively. Yellow nodes in the subnetworks of **(b)** and **(c)** highlight genes of highly clustered modules. Reference Additional file 5 for node names and gene relationships of the highly clustered modules in (b.) and (c.).

To identify pathways potentially involved in mediating transgenerational plastic germination responses, we used Cytoscape to assess and visualize the coexpression networks of genes responsive to parental exposure to salinity stress within the context of the *Arabidopsis* seed coexpression network [33]. This genome-wide co-expression network describes transcriptional interactions of dormant and germinating seeds that were derived from expression meta-data generated exclusively from mature *Arabidopsis* seeds (Figure 3). This network consists of 8,261 nodes (genes) that is comprised of distinct regions of clustered interaction enriched in transcripts identified in microarray data (Figure 3a). Outlined in yellow are defined regions in the network: region 1 represents clusters of genes associated with nongermination/dormancy; region 2 represents a transition from nongerminating/dormancy to germinating states; region 3 is associated with germination.

We queried the genes responsive to salinity stress in the *Arabidopsis* seed co-expression network among the four genotypes and found sub-networks associated with distinct regions of the seed germination network (Figure 3 a-d). No significant sub-networks were found for TN7.22 and TN8.22 saline responsive transcripts, which parallels the lack of transgenerational plasticity in germination behavior of these two genotypes. In contrast, we identified significant sub-networks for both TN1.13 and TN1.15 that are associated with regions that correspond to seed germination in the *Arabidopsis* seed network (Figure 3b and c, region 3). To extract more meaningful co-regulated gene interactions from the complex sub-networks (Figure 3b and c), we implemented MCODE to identify modules of tightly clustered co-expressed genes that represent groups of genes that are potentially functioning in the same biological pathway. We identified modules within the salt responsive sub-networks (Figure 3b and c, yellow nodes) and found clusters significantly enriched for GO terms associated with RNA metabolism, translation, and leaf development (Additional file 1).

Because TN1.13 and TN1.15 offspring exhibit similar patterns of parental environmental effects on germination response to salinity (Figure 1), we queried the overlapping salt responsive genes to identify the network relationship among these genes. Interestingly, the sub-network derived from these overlapping salt responsive genes correspond to regions of the seed network that are associated with seed dormancy (Figure 3d, region 1), supporting the shared delayed germination responses to salinity we observed in these two genotypes. Furthermore, among the shared salt responsive transcripts in TN1.13 and TN1.15 that are involved in the seed dormancy network (Figure 3d), we find genes associated with ABA up-regulation (Table 1) that account for over 30% of the dormancy related genes.

**Table 1:** **Genes associated with ABA up**-**regulation within the dormancy coexpression sub**-**network**

***A*** *.* ***thaliana*** **genes**	***M*** *.* ***truncatula*** **orthologs**	**Annotation**
At5g24670	Medtr8g103020	**TRNA Adenosine Deaminase 3**; involved in RNA editing
At5g62600	Medtr7g037000	**MOS14**; Nuclear importer of serine-arginine rich (SR) proteins; involved in the regulation of splicing R genes
At4g35800	Medtr5g023020	**NRPB1** DNA-directed RNA polymerase; DNA methylation, gene silencing by RNA, RNA splicing
At2g44200	Medtr2g028200	**CBF1**-interacting co-repressor; Pre-mRNA splicing factor
At3g54280	Medtr4g034920	**RGD3**-Root Growth Defective 3; Chromatin remodeling complex
At5g13300	Medtr7g020860	**SFC** (SCARFACE); involved in response to auxin
At2g38410	Medtr7g072310	**VHS** domain-containing protein / GAT domain-containing protein; involved in intracellular protein transport
At2g18700	Medtr4g129270	**ATTPS11**; Arabidopsis thaliana trehalose phosphatase/synthase 11; involved in trehalose biosynthesis
At2g39340	Medtr3g073080	**SAC3**/**GANP** family protein

## Discussion

The goal of this study is to test for genetic variation in transgenerational plastic germination behavior of four natural *M. truncatula* genotypes and identify stored seed transcripts that are in involved in salt response. This is the initial step to identify potential genes and molecular pathways that mediate germination response to salinity stress. We demonstrate genetic variation in transgenerational plasticity of germination timing upon parental exposure to salinity stress. The parental environment influences seed size in some genotypes, but there is no overall relationship between seed size and germination behavior. This suggests that parental environmental signals other than resource investment in seed size are important influences on germination timing. Our study shows genotype-dependent parental environmental effects on the expression level of stored seed transcripts. Among the transcripts responsive to parental exposure to salinity stress, we identified genes associated with seed dormancy pathway that may facilitate delayed germination response in saline conditions. This may reflect an adaptive salt avoidance strategy as seen in other pasture legumes [62]. Overall, our results suggest that transgenerational plasticity can play an ongoing role in adaptation to saline habitats in *M. truncatula* and identifies molecular pathways that may underlie the modulation of germination behavior under salt stress.

Parental environmental effects on seed size may reflect differential parental resource investment in offspring, a transgenerational mechanism that influences offspring development and performance. Previous studies have suggested that parental environmental effects on seed size influences germination timing [26,41,81]. In *Plantago lanceolata,* seed size variation induced by the parental environment during seed maturation is primarily driven by the seed coat rather than the embryo and endosperm [14,41,82]: heavier seeds tend to have heavier or thicker seed coats that delay germination response. In this study, where the seed coat was disrupted to focus on physiological dormancy, we find that seed size did not correlate with germination timing in any of the four genotypes. Instead we find that parental genotypes TN7.22 and TN8.22 exposed to salinity stress produced larger seeds that confer a growth advantage: plants originating from large seeds tend to develop larger leaves and produce more leaves in comparison to plants originating from smaller seeds (Table 2). These results suggest that *M. truncatula* genotypes respond to salinity stress by increasing resource investment into endosperm or embryo size rather than the seed coat. However, further studies in *M. truncatula* addressing the partitioning of parental investment to seed traits in response to environmental stress are necessary to understand how the parental environment shapes offspring development and response through resource provisioning to seeds.

**Table 2:** **Seed size correlation with performance traits**

	**Seed size**			
	**TN1.13**	**TN1.15**	**TN7.22**	**TN8.22**
**Leaf size**	0.05[−0.28,0.38]	0.13[−0.19,0.43]	0.61***[0.36,0.78]	0.58***[0.31,0.76]
**Number of leaves**	−0.18[−0.48,0.15]	−0.15[−0.17,0.44]	0.49**[0.21,0.70]	0.35*[0.04,0.6]

*P < 0.05; **P < 0.001; ***P < 0.0001.

Early germination and increased parental investment into seed size confers a growth advantage that may be indicative of salt tolerance rather than salt avoidance. Seeds from TN7.22 and TN8.22 parental plants exposed to salinity tended to germinate earlier in saline conditions irrespective of the offspring environment (Figures 1c and d), which could potentially be an unfavorable response in saline habitats. However, we find that early germination is correlated with higher growth potential in leaf size (Pearson correlation, r = −0.49, *P <* 0.0001) and number of leaves (Pearson correlation, r = −0.32, *P* < 0.0001). A previous study of Tunisian saline adapted genotypes showed that some genotypes exhibit a level of salt tolerance during germination by modulating metabolic and physiological processes to maintain ion balance in the root system [83] to improve water uptake important for photosynthesis and growth during salinity stress. Overall, we detect parental environmental effects on offspring performance that does not depend on the offspring, where some parental genotypes invest resources into seed size to optimize offspring performance in the next generation.

In contrast, we find that some parental genotypes influence offspring germination and seedling development to ensure seedling survival in saline conditions. Genotypes TN1.13 and TN1.15 displayed significant transgenerational plastic germination responses: offspring delayed germination in saline conditions only when parental plants were exposed to salinity stress. This may represent a viable salt avoidance mechanism to ensure seedling survival in saline environments. Because saline habitats experience high levels of salinity early in the rainy season and levels begin to dissipate with subsequent rain [60,84], pasture legumes adapted to these habitats have evolved delayed germination to avoid toxic levels of salinity early in the growing season [62,84]. In addition to germination, these genotypes also show transgenerational plasticity in the timing of unifoliate and first trifoliate development (Additional file 2, data not shown), suggesting that parental environmental effects persist past seedling establishment and into early seedling development. Further, there were no seed size effects on germination behavior and no significant seed size differences between parental environments (Figure 2, Additional file 4), suggesting that parental environmental signals other than resource investment in seed size may play a role in modulating germination timing in these two genotypes.

Because stored seed transcripts play a pivotal role in seed germination [53], we hypothesize that stored seed transcripts may represent a transgenerational mechanism some parental genotypes exploit to influence offspring germination behavior. We detected genotype-dependent parental environmental effects on the expression of stored seed transcripts, with some involved in seed dormancy and germination pathways (Figure 3). Previous studies have shown that the expression of specific stored seed transcripts influenced by parental exposure to stress is correlated with the magnitude of seed dormancy [35,49,50]. A recent study in *Arabidopsis* demonstrated that cold stress induced seed transcripts in dry, mature seeds were associated with genes that regulate seed dormancy and germination timing [35]. Because TN1.13 and TN1.15 offspring exhibit similar patterns of parental environmental effects on germination response to salinity (Figure 1), we queried the overlapping salt responsive genes to identify the network relationship among these genes. Interestingly, we identified a sub-network that corresponds to a region of the seed network associated with seed dormancy (Figure 3d, region 1), thus supporting the shared delayed germination responses to salinity observed in these two genotypes.

A plant’s ability to tolerate or adapt to salt stress often comes with a cost to vegetative growth and reproduction due to both genetic and resource limitations [10,85]. *M. trucatula* plants adapted to saline habitats are typically smaller and exhibit reduced reproductive output compared to plants adapted to non-saline habitats [57]. In our study, offspring exposed to saline conditions, on average, produced fewer leaves than offspring growing in non-saline conditions (Wilcoxon rank sum test, W = 16, *P* <0.05). Delayed germination is often correlated with reduced performance and competitive ability [86,87], while early seedling emergence is associated with increased fitness [88]. However, if early germination results in seedling death, then we would expect selection to favor delayed over early germination at the expense of reduced growth potential. Here we find that delayed germination is correlated with smaller leaf size (Pearson correlation, r = −0.32, *P* < 0.0001) and reduced number of leaves (Pearson correlation, r = −0.62, *P* <0.00001). This may be due to tradeoffs between survival and performance in stressful environments [89] Here we find that some genotypes may exhibit this tradeoff in germination timing and this is likely due to genotypic differences in the degree of salt tolerance and parental strategy to cope with salinity stress.

Despite the fitness cost of delayed germination, dormancy mechanisms have evolved as an adaptive response to environmental uncertainty across many species of plants [90]. Germination timing is an important life history trait that determines the environment of the developing plant; thus, influences seedling survival and adaptive traits later in life [29]. After-ripened non-dormant seeds can induce secondary dormancy when environmental conditions are unfavorable [91,92]. ABA is a pleiotropic plant hormone, playing key roles in a variety of developmental pathways that include seed development and dormancy [93], in addition to adaptive stress responses to environmental perturbations in plants [94-96]. Numerous studies have observed ABA-mediated gene expression in response to drought and salt stress [97]. In our study, among the shared salt responsive transcripts in TN1.13 and TN1.15, we find transcripts associated with ABA up-regulation (Table 1) and these transcripts are involved in the seed dormancy network (Figure 3d). These ABA related genes account for 30% of the dormancy related genes and they are functionally characterized to be involved in post-transcriptional regulation (TRNA Adenosine Deaminase 3, MOS14, NRPBI, CBF1), epigenetic mechanisms (NRPB1, RGD3) and growth and developmental processes (SFC; Table 1; Figure 3d). This suggests a dynamic crosstalk between ABA and gene regulatory pathways that potentially regulate transgenerational plasticity in germination timing under parental salinity stress. Although phytohormone ABA does not appear to be directly involved in secondary dormancy, transcription factors regulated by ABA in dry, mature seeds may be involved in transitioning after-ripened non-dormant seeds to secondary dormancy [35,42]. Although our results suggest that seeds may delay germination through the induction of secondary dormancy, but it is unclear whether *M. truncatula* seeds undergo secondary dormancy.

Recently, the transmission of epigenetic marks regulating gene expression (i.e., DNA methylation) has emerged as a candidate mechanism mediating adaptive transgenerational responses in plants [98,99]. In fact, we found significant enrichment of seed transcripts associated with chromatin remodeling and miRNA production (Additional file 1), which is consistent with the results observed in *Arabidopsis* seed transcriptome [74]. Although these processes have been documented to mediate transgenerational effects through DNA methylation [100,101], these mechanisms were not implicated in our study as mediating salt response in the seed transcriptome. However, our results do not discount the possibility that epigenetic mechanisms play a role in mediating transgenerational effects, because our experiment was not designed to capture the influence of these processes in response to salinity stress. Therefore, bisulfite and miRNA sequencing in future experiments will be necessary to understand the relationship between epigenetic mechanism and transgenerational plasticity in germination behavior.

Post-transcriptional processing of stored seed transcripts play a critical role in seed germination [53], and are likely involved in modulating germination behavior when parental plants are exposed to salinity stress. In this study, we find that alternative splicing and translation processes respond to parental exposure to salinity stress (Additional file 5, reference Additional file 6 for the complete list of genes corresponding to salt responsive transcripts), which might represent novel mechanisms mediating transgenerational plasticity in seedling development. Because translation of stored seed transcripts represent a critical step in seed germination [53], it is not surprising that we find post-transcriptional regulatory networks involved in responding to parental exposure to salinity stress. Genome-wide post-transcriptional regulation under abiotic stress conditions has shown to play an important role in translational regulation of stress responsive transcripts [102]. The amount of specific proteins translated is critical for biological pathways to function optimally in response to environmental perturbations. Furthermore, different alternative splice variants of transcription factors in response to environmental cues are involved in regulating seed dormancy and germination in *Arabidopsis* [48,103]. Since TN1.13 and TN1.15 early offspring response to salinity depends on both the parental and offspring environment, alternative splicing and translational regulation may represent a viable transgenerational mechanism that can process parental environmental cues to influence offspring response to salinity stress.

## Conclusion

In this study, we have demonstrated that parental environmental signals can be transmitted through the expression of stored seed transcripts or resources provisioned to the seed to influence offspring response and development under salinity stress. Parental environmental effects on seed dormancy permits temporal escape from unfavorable conditions early in the germination season, while parental investment in larger seeds provide seedlings with resources to improve performance under unfavorable conditions. Although the mature seed transcriptome has been characterized in several plant species [74] including *M. truncatula* seeds [66], none of these studies have linked the effect of stored seed transcripts to transgenerational plastic germination behavior. Our study demonstrates a potential for parental control over seed dormancy by influencing the expression of stored seed transcripts and propose novel post-transcriptional mechanisms involved in germination under salinity stress. Furthermore, the genotypic differences seen in parental environmental effects on the expression of stored seed transcripts and offspring germination response suggest that transgenerational plasticity of germination behavior can potentially evolve under saline conditions.

### Availability of supporting data

The data set supporting the results of this article is available in the NCBI Short Read Archive under SRP012122 and provided as supplementary data.

## Additional files

Additional file 1:
**Complete list of significant gene ontology terms for total genes expressed in the seed transcriptome enriched in biological pathways.**
Additional file 2:
**ANOVA P-values for offspring traits explained by genotype (G), parental environment (PE), offspring environment (OE) and the interaction terms, with seed weight as a covariate for age of germination, age of unifoliate and first trifoliate development.**
Additional file 3:
**ANOVA F-values for offspring germination timing explained by the parental environment (PE), offspring environment (OE) and all possible interactions with seed mass as a covariate for each genotype. Bold values indicate significant effects.** Df: degrees of freedom Res. Df: residual degrees of freedom. Bonferroni correction for multiple comparisons *P* < 0.0125. Additional file 4:
**ANOVA on seed weight (g) differences between 0 mM and 100 mM parental environment for each genotype.** Bonferroni correction for multiple comparisons *P* < 0.0125. Additional file 5:
**Discrete tightly clustered modules representing putative biological pathways.** (a.) MCODE cluster identified from TN1.13 subnetwork (Figure 3b) and TN1.15 subnetwork (Figure 3c). (b.) Functional interactions between genes associated with significant overrepresented GO terms. Additional file 6:
**Salt responsive gene annotations of** ***Arabidopsis thaliana*** **and** ***M. truncatula*** orthologs. The genes listed below are salt responsive transcripts that are significantly associated with sub-networks in the SeedNet for TN1.13, TN1.15 and the overlapping transcripts.
