# Supplementary material for: Genetic variation of transgenerational plasticity of offspring germination in response to salinity stress and the seed transcriptome of Medicago truncatula
Source: BMC Evol Biol. 2015 Apr 1;15:59. doi: 10.1186/s12862-015-0322-4 (PMC4406021; doi:10.1186/s12862-015-0322-4)
Supplement: Additional file 1: — Complete list of significant gene ontology terms for total genes expressed in the seed transcriptome enriched in biological pathways. [file 12862_2015_322_MOESM1_ESM.doc]

**Additional file 1.** Significant Gene Ontology terms for the seed transcriptome.

| **Gene Ontology** | **Biological Process** | **P-value** |
| --- | --- | --- |
| GO:0044260 | Cellular macromolecule metabolic process | 7.38e-34 |
| **GO:0010467** | **Gene expression** | **6.97e-29** |
| GO:0044265 | Cellular macromolecule catabolic process | 6.22e-28 |
| GO:0044238 | Primary metabolic process | 7.29e-27 |
| GO:0006508 | Proteolysis | 2.63e-21 |
| GO:0015031 | Protein transport | 3.77e-21 |
| GO:0019941 | Modification-dependent protein catabolic process | 4.57e-20 |
| **GO:0006412** | **Translation** | **4.05e-13** |
| GO:0022613 | Ribonucleoprotein complex biogenesis and assembly | 3.37e-11 |
| GO:0016070 | **RNA metabolic process** | **6.25e-11** |
| GO:0006970 | **Response to osmotic stress** | **3.57e-08** |
| GO:0006396 | **RNA processing** | **6.23e-07** |
| GO:0009657 | Plastid organization and biogenesis | 1.28e-06 |
| GO:0006807 | Nitrogen compound metabolic process | 1.84e-06 |
| GO:0006325 | **Establishment and maintenance of chromatin architecture** | **5.38e-06** |
| GO:0007275 | Multicellular organismal development | 3.05e-05 |
| GO:0009628 | **Response to abiotic stimulus** | **3.70e-05** |
| GO:0006464 | Protein modification process | 6.28e-05 |
| GO:0044419 | Interspecies interaction between organisms | -7.45e-05 |
| GO:0009791 | Post-embryonic development | 7.62e-05 |
| GO:0051276 | **Chromosome organization and biogenesis** | **7.72e-05** |
| GO:0009877 | Nodulation | -9.72e-05 |
| GO:0044403 | Symbiosis encompassing mutualism through parasitism | -1.09e-04 |
| GO:0022414 | Reproductive process | 3.76e-04 |
| GO:0016568 | **Chromatin modification** | 6.86e-04 |
| GO:0008380 | **RNA splicing** | 6.86e-04 |
| GO:0032774 | **RNA biosynthetic process** | 8.97e-04 |
| GO:0006351 | **Transcription DNA-dependent** | 8.97e-04 |
| GO:0019725 | **Cellular homeostasis** | 1.67e-04 |
| GO:0035196 | **miRNA-mediated gene silencing production of miRNAs** | 1.72e-03 |
| GO:0000375 | **RNA splicing via transesterification reactions** | 6.50e-03 |
| GO:0042221 | Response to chemical stimulus | 8.32e-03 |
| GO:0044271 | Nitrogen compound biosynthetic process | 8.99e-03 |
| **GO:0016071** | **mRNA metabolic process** | **0.0139** |
| GO:0006457 | Protein folding | 0.0143 |
| GO:0031047 | **RNA-mediated gene silencing** | 0.0160 |
| GO:0006355 | Regulation of transcription DNA-dependent | 0.0202 |
| GO:0009887 | Organ morphogenesis | 0.0207 |
| GO:0009266 | Response to temperature stimulus | 0.0278 |
| GO:0006338 | **Chromatin remodeling** | 0.0278 |
| GO:0043487 | Regulation of RNA stability | 0.0283 |
| GO:0006402 | mRNA catabolic process | 0.0283 |
| GO:0006399 | tRNA metabolic process | 0.0304 |
| GO:0006413 | **Translational initiation** | 0.0353 |
