# Supplementary material for: Genetic variation of transgenerational plasticity of offspring germination in response to salinity stress and the seed transcriptome of Medicago truncatula
Source: BMC Evol Biol. 2015 Apr 1;15:59. doi: 10.1186/s12862-015-0322-4 (PMC4406021; doi:10.1186/s12862-015-0322-4)
Supplement: Additional file 2: — ANOVA P-values for offspring traits explained by genotype (G), parental environment (PE), offspring environment (OE) and the interaction terms, with seed weight as a covariate for age of germination, age of unifoliate and first trifoliate development. [file 12862_2015_322_MOESM2_ESM.doc]

**Additional file 2.** ANOVA table of P-values.

|  | Age at germination | Age at unifoliate dev. | Age at trifoliate dev. |
| --- | --- | --- | --- |
| G | 0.3325 | 0.6322 | 0.9837 |
| PE | 0.2479 | 0.3300 | 0.3819 |
| OE | **0.0097** | **0.0008** | **0.0236** |
| Seed weight | 0.9494 | 0.3253 | 0.1751 |
| G x PE | 0.2027 | 0.3209 | 0.4080 |
| G x OE | 0.0679 | 0.6124 | 0.6907 |
| PE x OE | **0.0006** | **0.0030** | **0.0146** |
| G x PE x OE | **0.0002** | **0.0172** | **0.0256** |
| Adjusted-R^2^ | 0.54 | 0.55 | 0.42 |
