# Supplementary material for: Genetic variation of transgenerational plasticity of offspring germination in response to salinity stress and the seed transcriptome of Medicago truncatula
Source: BMC Evol Biol. 2015 Apr 1;15:59. doi: 10.1186/s12862-015-0322-4 (PMC4406021; doi:10.1186/s12862-015-0322-4)
Supplement: Additional file 3: — ANOVA F-values for offspring germination timing explained by the parental environment (PE), offspring environment (OE) and all possible interactions with seed mass as a covariate for each genotype. Bold values indicate significant effects. Df: degrees of freedom Res. Df: residual degrees of freedom. Bonferroni correction for multiple comparisons P < 0.0125. [file 12862_2015_322_MOESM3_ESM.doc]

**Additional file 3.** ANOVA table of F-values. Bonferroni corrected *P* < 0.0125.

|  | TN1.13 | TN1.15 | TN7.22 | TN8.22 |
| --- | --- | --- | --- | --- |
| PE | 0.297 | 1.377 | 0.132 | 0.062 |
| OE | **46.060***** | **32.638***** | **48.364***** | **32.042***** |
| PExOE | **9.366**** | **19.964***** | 0.297 | 3.107 |
| Seed mass | 0.422 | 0.751 | 0.325 | 0.121 |
| Df/Res. Df | 1/32 | 1/34 | 1/34 | 1/35 |

**P* < 0.0125 ***P* < 0.001 ****P* < 0.0001
