# Supplementary material for: Genetic variation of transgenerational plasticity of offspring germination in response to salinity stress and the seed transcriptome of Medicago truncatula
Source: BMC Evol Biol. 2015 Apr 1;15:59. doi: 10.1186/s12862-015-0322-4 (PMC4406021; doi:10.1186/s12862-015-0322-4)
Supplement: Additional file 4: — ANOVA on seed weight (g) differences between 0 mM and 100 mM parental environment for each genotype. Bonferroni correction for multiple comparisons P < 0.0125. [file 12862_2015_322_MOESM4_ESM.doc]

**Additional file 4.** ANOVA F-values and linear regression coefficients seed size and germination timing. Bonferroni corrected *P* < 0.0125

|  | F-value | Coefficient | P-value |
| --- | --- | --- | --- |
| TN1.13 | 3.85_(138)_ | 3.85 | 0.06 |
| TN1.15 | 6.01_(138)_ | 6.01 | 0.02 |
| TN7.22 | 254.5_(138)_ | 254.51 | **2.2e-16** |
| TN8.22 | 9.37_(1.38)_ | 9.37 | **0.004** |
