# Supplementary figures and images for: Genetic variation of transgenerational plasticity of offspring germination in response to salinity stress and the seed transcriptome of Medicago truncatula
Source: BMC Evol Biol. 2015 Apr 1;15:59. doi: 10.1186/s12862-015-0322-4 (PMC4406021; doi:10.1186/s12862-015-0322-4)

**Additional file 5.** Coexpression modules of TN1.13 and TN1.15 salt responsive transcripts.


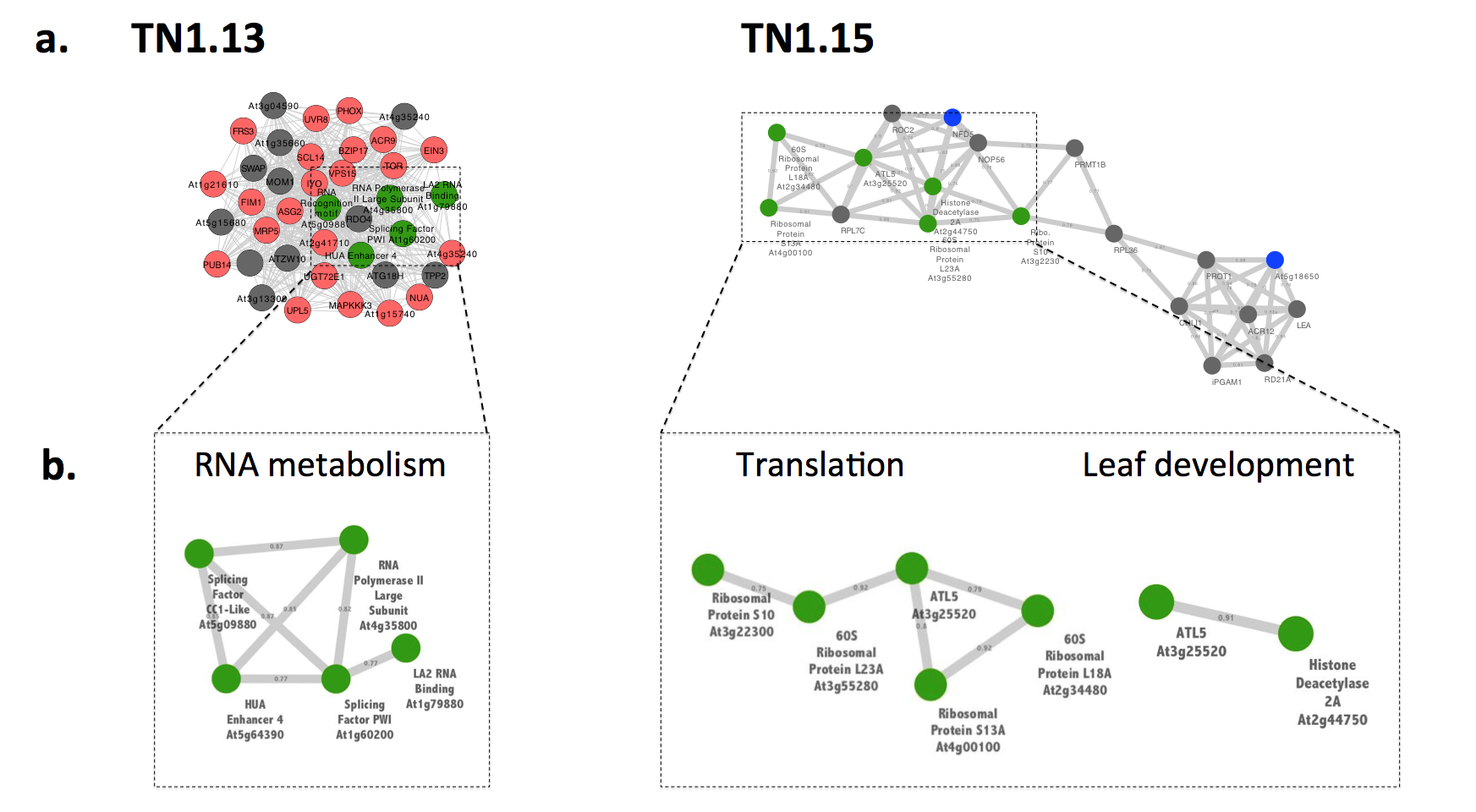

Supplement: Additional file 5: — Discrete tightly clustered modules representing putative biological pathways. (a.) MCODE cluster identified from TN1.13 subnetwork (Figure 3b) and TN1.15 subnetwork (Figure 3c). (b.) Functional interactions between genes associated with significant overrepresented GO terms. [file 12862_2015_322_MOESM5_ESM.doc]
