# Supplementary material for: Genetic variation of transgenerational plasticity of offspring germination in response to salinity stress and the seed transcriptome of Medicago truncatula
Source: BMC Evol Biol. 2015 Apr 1;15:59. doi: 10.1186/s12862-015-0322-4 (PMC4406021; doi:10.1186/s12862-015-0322-4)
Supplement: Additional file 6: — Salt responsive gene annotations of Arabidopsis thaliana and M. truncatula orthologs. The genes listed below are salt responsive transcripts that are significantly associated with sub-networks in the SeedNet for TN1.13, TN1.15 and the overlapping transcripts. [file 12862_2015_322_MOESM6_ESM.doc]

**Additional file 6. List of *M. truncatula* and *Arabidopsis* orthologs.**

| **AT** | **MTR** | **Annotation** | **Type** |
| --- | --- | --- | --- |
| AT1G78580 | Medtr8g087910 Medtr8g087930 | ATTPS1 (TREHALOSE-6-PHOSPHATE SYNTHASE); transferase transferring glycosyl groups | Overlap |
| AT2G18700 | Medtr4g129270 | ATTPS11 (Arabidopsis thaliana trehalose phosphatase/synthase 11); transferase transferring glycosyl groups | Overlap |
| AT3G08510 | Medtr3g069280 Medtr3g070720 Medtr5g071010 Medtr5g082620 | ATPLC2 (PHOSPHOLIPASE C 2); phospholipase C | Overlap |
| AT3G12940 | Medtr2g069480 | similar to unknown protein "Arabidopsis thaliana" (TAIR:AT3G19895.1); similar to unnamed protein product "Vitis vinifera" (GB:CAO23819.1) | Overlap |
| AT3G14050 | Medtr8g020880 | RSH2 (RELA-SPOT HOMOLOG); catalytic | Overlap |
| AT3G26720 | Medtr7g084040 | glycosyl hydrolase family 38 protein | Overlap |
| AT4G18880 | Medtr1g106840 Medtr8g087540 | AT-HSFA4A (Arabidopsis thaliana heat shock transcription factor A4A); DNA binding / transcription factor | Overlap |
| AT4G35580 | Medtr3g093040 | no apical meristem (NAM) family protein | Overlap |
| AT5G01670 | Medtr7g070500 | aldose reductase putative | Overlap |
| AT5G24670 | Medtr8g103020 | hydrolase/ zinc ion binding | Overlap |
| AT5G51130 | Medtr8g102950 | contains InterPro domain Methyltransferase type 12 (InterPro:IPR013217); contains InterPro domain Bicoid-interacting 3 (InterPro:IPR010675) | Overlap |
| AT5G54730 | Medtr2g082770 | AtATG18f (Arabidopsis thaliana homolog of yeast autophagy 18 (ATG18) f) | Overlap |
| AT5G65280 | Medtr3g086910 | GCL1 (GCR2-LIKE 1); catalytic | Overlap |
| ATMG00640 | Medtr6g042930 Medtr6g043020 | encodes a plant b subunit of mitochondrial ATP synthase based on structural similarity and the presence in the F(0) complex. | Overlap |
| AT1G02060 | Medtr7g044790 | pentatricopeptide (PPR) repeat-containing protein | TN113 |
| AT1G04120 | Medtr7g098690 | ATMRP5 (Arabidopsis thaliana multidrug resistance-associated protein 5) | TN113 |
| AT1G06570 | Medtr5g090980 | PDS1 (PHYTOENE DESATURATION 1) | TN113 |
| AT1G07510 | Medtr1g044600  Medtr4g064350 | FTSH10 (FtsH protease 10); ATPase | TN113 |
| AT1G07530 | Medtr2g097310 Medtr2g097350 Medtr2g097390 Medtr4g064160 | scarecrow-like transcription factor 14 (SCL14) | TN113 |
| AT1G08460 | Medtr2g087270 | HDA08 (histone deacetylase 8); histone deacetylase | TN113 |
| AT1G08570 | Medtr7g085310 | thioredoxin family protein | TN113 |
| AT1G11020 | Medtr4g113520 | zinc finger (C3HC4-type RING finger) family protein | TN113 |
| AT1G11650 | Medtr4g131970 | ATRBP45B; RNA binding | TN113 |
| AT1G15740 | Medtr3g043810 Medtr4g009870 Medtr4g116170 | leucine-rich repeat family protein | TN113 |
| AT1G23070 | Medtr2g042580 | similar to unknown protein "Arabidopsis thaliana" (TAIR:AT4G38360.2); similar to unnamed protein product "Vitis vinifera" (GB:CAO65220.1); contains InterPro domain Protein of unknown function DUF300 (InterPro:IPR005178) | TN113 |
| AT1G26665 | Medtr8g085570 | similar to RNA polymerase II mediator complex protein-related | TN113 |
| AT1G27320 | Medtr3g085130 | AHK3 (ARABIDOPSIS HISTIDINE KINASE 3) | TN113 |
| AT1G32130 | Medtr5g028030 | similar to IWS1 C-terminus family protein | TN113 |
| AT1G35190 | Medtr8g102600 | oxidoreductase 2OG-Fe(II) oxygenase family protein | TN113 |
| AT1G35660 | Medtr1g050670 | binding | TN113 |
| AT1G36990 | Medtr5g060280 | similar to unnamed protein product "Vitis vinifera" | TN113 |
| AT1G47970 | Medtr4g122830 | unknown protein | TN113 |
| AT1G48280 | Medtr4g124070 | hydroxyproline-rich glycoprotein family protein | TN113 |
| AT1G50030 | Medtr5g005380 | TOR (TARGET OF RAPAMYCIN) | TN113 |
| AT1G53570 | Medtr3g096230 Medtr8g093730 | MAP3KA (Mitogen-activated protein kinase kinase kinase 3); kinase | TN113 |
| AT1G54710 | Medtr1g082300 | AtATG18h (Arabidopsis thaliana homolog of yeast autophagy 18 (ATG18) h) | TN113 |
| AT1G55325 | Medtr3g083500 | similar to hypothetical protein OsI_019475 "Oryza sativa (indica cultivar-group)" | TN113 |
| AT1G60200 | Medtr5g034430 | splicing factor PWI domain-containing protein / RNA recognition motif (RRM)-containing protein | TN113 |
| AT1G62290 | Medtr4g132420 | aspartyl protease family protein | TN113 |
| AT1G62710 | Medtr4g101730 | BETA-VPE (vacuolar processing enzyme beta); cysteine-type endopeptidase | TN113 |
| AT1G65950 | Medtr5g077080 | ABC1 family protein | TN113 |
| AT1G69220 | Medtr5g045190 | SIK1 (ERINE/THREONINE KINASE 1); kinase | TN113 |
| AT1G75440 | Medtr5g021260  Medtr8g076490 | UBC16 (UBIQUITIN-CONJUGATING ENZYME 16); ubiquitin-protein ligase | TN113 |
| AT1G76490 | Medtr5g024880 | HMG1 (3-HYDROXY-3-METHYLGLUTARYL COA REDUCTASE) | TN113 |
| AT1G78580 | Medtr8g087910 Medtr8g087930 | ATTPS1 (TREHALOSE-6-PHOSPHATE SYNTHASE); transferase transferring glycosyl groups | TN113 |
| AT2G02370 | Medtr2g028450 Medtr3g064100 | similar to unnamed protein product "Vitis vinifera" (GB:CAO22255.1); contains InterPro domain SNARE associated Golgi protein (InterPro:IPR015414) | TN113 |
| AT2G17510 | Medtr8g073170 Medtr8g073450 | EMB2763 (EMBRYO DEFECTIVE 2763); RNA binding / ribonuclease | TN113 |
| AT2G32900 | Medtr5g084830 | ATZW10 | TN113 |
| AT2G35800 | Medtr1g095780 | mitochondrial substrate carrier family protein | TN113 |
| AT2G36350 | Medtr3g010260 Medtr7g104180 | protein kinase putative | TN113 |
| AT2G39190 | Medtr1g095350 Medtr1g095480 | ATATH8 (ABC2 homolog 8) | TN113 |
| AT2G39570 | Medtr2g096640 Medtr7g024320 | ACT domain-containing protein | TN113 |
| AT2G41710 | Medtr7g091390 | ovule development protein putative | TN113 |
| AT2G42520 | Medtr3g083690 | DEAD box RNA helicase putative | TN113 |
| AT2G44710 | Medtr7g081210 | RNA recognition motif (RRM)-containing protein | TN113 |
| AT2G45600 | Medtr8g035520 Medtr8g035540 | hydrolase | TN113 |
| AT2G45910 | Medtr7g077780 | protein kinase family protein / U-box domain-containing protein | TN113 |
| AT3G02290 | Medtr7g016840 | zinc finger (C3HC4-type RING finger) family protein | TN113 |
| AT3G02750 | Medtr7g021530 Medtr7g080170 | protein phosphatase 2C family protein / PP2C family protein | TN113 |
| AT3G02860 | Medtr5g077800 | zinc ion binding | TN113 |
| AT3G04590 | Medtr1g079780 Medtr7g116320 | DNA-binding family protein | TN113 |
| AT3G06480 | Medtr2g032630 | DEAD box RNA helicase putative | TN113 |
| AT3G06670 | Medtr2g034560 Medtr4g121890 | binding | TN113 |
| AT3G07890 | Medtr5g098140 | RabGAP/TBC domain-containing protein | TN113 |
| AT3G10690 | Medtr1g031690 | DNA gyrase subunit A family protein | TN113 |
| AT3G11760 | Medtr1g056180 | similar to unknown protein "Arabidopsis thaliana" (TAIR:AT5G04860.1); similar to unnamed protein product "Vitis vinifera" (GB:CAO39924.1) | TN113 |
| AT3G15351 | Medtr4g093680 | similar to hypothetical protein OsI_025123 "Oryza sativa (indica cultivar-group)" (GB:EAZ03891.1) | TN113 |
| AT3G16830 | Medtr4g120900 | TPR2 (TOPLESS-RELATED 2) | TN113 |
| AT3G17970 | Medtr8g107280 | ATTOC64-III (ARABIDOPSIS THALIANA TRANSLOCON AT THE OUTER MEMBRANE OF CHLOROPLASTS 64-III); binding / carbon-nitrogen ligase with glutamine as amido-N-donor | TN113 |
| AT3G19240 | Medtr4g086240 | similar to dem protein-related / defective embryo and meristems protein-related | TN113 |
| AT3G20720 | Medtr2g082890 Medtr2g082910 Medtr4g081300 | similar to hypothetical protein OsI_016901 "Oryza sativa (indica cultivar-group)" (GB:EAY95668.1); similar to predicted protein "Physcomitrella patens subsp. patens" (GB:EDQ56411.1) | TN113 |
| AT3G20770 | Medtr5g087790 | EIN3 (ETHYLENE-INSENSITIVE3); transcription factor | TN113 |
| AT3G22490 | Medtr1g072090 Medtr2g076230 | late embryogenesis abundant protein putative / LEA protein putative | TN113 |
| AT3G26720 | Medtr7g084040 | glycosyl hydrolase family 38 protein | TN113 |
| AT3G51370 | Medtr8g074930 | protein phosphatase 2C putative / PP2C putative | TN113 |
| AT3G51620 | Medtr5g075280 | similar to nucleotidyltransferase family protein "Arabidopsis thaliana" (TAIR:AT3G56320.1; contains domain TOPOISOMERASE-RELATED PROTEIN (PTHR23092) | TN113 |
| AT3G52850 | Medtr7g073350 Medtr8g005800 | ATELP/ATELP1/BP-80/BP80/BP80B/VSR-1/VSR1 (ARABIDOPSIS THALIANA EPIDERMAL GROWTH FACTOR RECEPTOR-LIKE PROTEIN) | TN113 |
| AT3G54280 | Medtr4g034920 | ATP binding / DNA binding / helicase | TN113 |
| AT3G54850 | Medtr1g093950 | armadillo/beta-catenin repeat family protein / U-box domain-containing family protein | TN113 |
| AT3G58050 | Medtr7g093630 | similar to unknown protein "Arabidopsis thaliana" (TAIR:AT2G41960.1) | TN113 |
| AT3G62770 | Medtr1g083230 Medtr7g108520 | AtATG18a (Arabidopsis thaliana homolog of yeast autophagy 18 (ATG18) a) | TN113 |
| AT4G02020 | Medtr1g086980 Medtr7g109560 Medtr7g055680 | EZA1 (SWINGER); transcription factor | TN113 |
| AT4G04670 | Medtr4g132120 | Met-10 like family protein / kelch repeat-containing protein | TN113 |
| AT4G05420 | Medtr8g089380 | DDB1A (UV-damaged DNA-binding protein 1A); DNA binding | TN113 |
| AT4G20850 | Medtr1g101030 | TPP2 (TRIPEPTIDYL PEPTIDASE II); subtilase | TN113 |
| AT4G21710 | Medtr1g023500 | NRPB2 (EMBRYO DEFECTIVE 1989); DNA binding | TN113 |
| AT4G26700 | Medtr5g094160 | ATFIM1 (Arabidopsis thaliana fimbrin 1); actin binding | TN113 |
| AT4G32160 | Medtr3g106770 | phox (PX) domain-containing protein | TN113 |
| AT4G32551 | Medtr1g011610 Medtr4g113080 Medtr3g107910 Medtr1g082650 | LUG (LEUNIG) | TN113 |
| AT4G32940 | Medtr1g016590 | GAMMA-VPE (Vacuolar processing enzyme gamma); cysteine-type endopeptidase | TN113 |
| AT4G35240 | Medtr3g104120Medtr5g098980 Medtr7g032230 | similar to unknown protein "Arabidopsis thaliana" (TAIR:AT2G17110.1) | TN113 |
| AT4G35580 | Medtr3g093040 | no apical meristem (NAM) family protein | TN113 |
| AT4G35800 | Medtr5g023020 Medtr5g077530 | NRPB1 (RNA POLYMERASE II LARGE SUBUNIT); DNA binding / DNA-directed RNA polymerase | TN113 |
| AT4G36980 | Medtr5g017430 | similar to unnamed protein product "Vitis vinifera" (GB:CAO71849.1); contains domain PTHR13161 (PTHR13161) | TN113 |
| AT5G01990 | Medtr7g074190 | auxin efflux carrier family protein | TN113 |
| AT5G06210 | Medtr3g025880 | RNA-binding protein putative | TN113 |
| AT5G09880 | Medtr3g095230 Medtr8g092550 | RNA recognition motif (RRM)-containing protein | TN113 |
| AT5G10730 | Medtr3g105660 | binding / catalytic/ coenzyme binding | TN113 |
| AT5G10860 | Medtr5g076010 Medtr5g076080 | CBS domain-containing protein | TN113 |
| AT5G10940 | Medtr3g100690 | transducin family protein / WD-40 repeat family protein | TN113 |
| AT5G13300 | Medtr7g020860 | SFC (SCARFACE) | TN113 |
| AT5G16110 | Medtr6g018430 Medtr6g084470 | similar to unknown protein "Arabidopsis thaliana" (TAIR:AT3G02555.1); similar to hypothetical protein "Cleome spinosa" (GB:ABD96917.1) | TN113 |
| AT5G16210 | Medtr7g082650 | HEAT repeat-containing protein | TN113 |
| AT5G16880 | Medtr4g077940 Medtr5g007480 Medtr4g078040 | VHS domain-containing protein / GAT domain-containing protein | TN113 |
| AT5G19330 | Medtr1g104870 Medtr5g005940 | armadillo/beta-catenin repeat family protein / BTB/POZ domain-containing protein | TN113 |
| AT5G22220 | Medtr4g052000 | E2F1; transcription factor | TN113 |
| AT5G35200 | Medtr4g115420 | epsin N-terminal homology (ENTH) domain-containing protein | TN113 |
| AT5G35930 | Medtr8g035620 | AMP-dependent synthetase and ligase family protein | TN113 |
| AT5G39510 | Medtr6g012860 Medtr7g011570 | ATVTI11/ATVTI1A/SGR4/VTI11/VTI1A/ZIG (VESICLE TRANSPORT V-SNARE 11); receptor | TN113 |
| AT5G46840 | Medtr5g029900 | RNA recognition motif (RRM)-containing protein | TN113 |
| AT5G49710 | Medtr5g065930 | similar to unknown protein "Arabidopsis thaliana" (TAIR:AT4G24590.1); similar to unnamed protein product "Vitis vinifera" (GB:CAO61623.1) | TN113 |
| AT5G51130 | Medtr8g102950 | contains InterPro domain Methyltransferase type 12 (InterPro:IPR013217); contains InterPro domain Bicoid-interacting 3 (InterPro:IPR010675) | TN113 |
| AT5G52660 | Medtr7g038290 | myb family transcription factor | TN113 |
| AT5G63860 | Medtr3g096780 Medtr8g094410 Medtr8g094450 | UVR8 (UVB-RESISTANCE 8) | TN113 |
| AT5G65530 | Medtr5g031870 | protein kinase putative | TN113 |
| ATMG00640 | Medtr1g107010 | encodes a plant b subunit of mitochondrial ATP synthase based on structural similarity and the presence in the F(0) complex. | TN113 |
| AT1G04300 | Medtr1g044450 | similar to meprin and TRAF homology domain-containing protein / MATH domain-containing protein | TN115 |
| AT1G08660 | Medtr7g084010 | glycosyl transferase family 29 protein / sialyltransferase family protein | TN115 |
| AT1G09780 | Medtr7g074570 | 23-biphosphoglycerate-independent phosphoglycerate mutase putative / phosphoglyceromutase putative | TN115 |
| AT1G09960 | Medtr5g067470 | SUT4 (SUCROSE TRANSPORTER 4); carbohydrate transmembrane transporter/ sucrose transmembrane transporter/ sucrose:hydrogen symporter/ sugar:hydrogen ion symporter | TN115 |
| AT1G10490 | Medtr2g028030 Medtr8g092060 | similar to predicted protein "Physcomitrella patens subsp. patens"; similar to predicted protein "Nematostella vectensis"; contains InterPro domain Protein of unknown function DUF699 ATPase putative (InterPro:IPR007807); contains InterPro domain Region of unknown function DUF1726 (InterPro:IPR013562) | TN115 |
| AT1G24510 | Medtr3g086330 | T-complex protein 1 epsilon subunit putative / TCP-1-epsilon putative / chaperonin putative | TN115 |
| AT1G25380 | Medtr5g055410 | mitochondrial substrate carrier family protein | TN115 |
| AT1G31812 | Medtr5g026740 | ACBP (ACYL-COA-BINDING PROTEIN); acyl-CoA binding | TN115 |
| AT1G32360 | Medtr8g066730 | zinc finger (CCCH-type) family protein | TN115 |
| AT1G47128 | Medtr1g018840 | RD21 (RESPONSIVE TO DEHYDRATION 21); cysteine-type peptidase | TN115 |
| AT1G56110 | Medtr4g103790 | NOP56 (ARABIDOPSIS HOMOLOG OF NUCLEOLAR PROTEIN NOP56) | TN115 |
| AT1G65930 | Medtr2g062840 Medtr5g077070 | isocitrate dehydrogenase putative / NADP isocitrate dehydrogenase putative | TN115 |
| AT1G73230 | Medtr3g020520 Medtr3g020660 Medtr4g071000 | nascent polypeptide-associated complex (NAC) domain-containing protein | TN115 |
| AT1G78290 | Medtr5g064540 | serine/threonine protein kinase putative | TN115 |
| AT1G80530 | Medtr2g104250 Medtr4g116210 | nodulin family protein | TN115 |
| AT2G05990 | Medtr4g074810 Medtr4g074950 | MOD1 (MOSAIC DEATH 1); enoyl-"acyl-carrier-protein" reductase (NADH)/ oxidoreductase | TN115 |
| AT2G16460 | Medtr5g021410 | similar to unknown protein "Arabidopsis thaliana" (TAIR:AT3G51090.1); similar to Os06g0713100 "Oryza sativa (japonica cultivar-group)" | TN115 |
| AT2G20450 | Medtr1g075720 Medtr4g120770 | 60S ribosomal protein L14 (RPL14A) | TN115 |
| AT2G25110 | Medtr3g106130 Medtr3g106160 Medtr1g012520 Medtr3g107040 Medtr3g107280 Medtr4g114650 | SDF2 (STROMAL CELL-DERIVED FACTOR 2-LIKE PROTEIN PRECURSOR) | TN115 |
| AT2G25870 | Medtr1g007320 Medtr1g007330 | haloacid dehalogenase-like hydrolase family protein | TN115 |
| AT2G32060 | Medtr4g006830 Medtr5g012890 Medtr8g093770 | 40S ribosomal protein S12 (RPS12C) | TN115 |
| AT2G34480 | Medtr2g019720 Medtr2g098000 Medtr5g091120 | 60S ribosomal protein L18A (RPL18aB) | TN115 |
| AT2G38770 | Medtr1g039070 | EMB2765 (EMBRYO DEFECTIVE 2765) | TN115 |
| AT2G39890 | Medtr3g069960 Medtr5g081630 | ProT1 (PROLINE TRANSPORTER 1); amino acid transmembrane transporter | TN115 |
| AT2G40010 | Medtr3g108280 Medtr5g082180 Medtr7g022400 | 60S acidic ribosomal protein P0 (RPP0A) | TN115 |
| AT2G41900 | Medtr3g029590 Medtr7g092070 | zinc finger (CCCH-type) family protein | TN115 |
| AT2G44060 | Medtr5g088850 | late embryogenesis abundant family protein / LEA family protein | TN115 |
| AT2G44120 | Medtr1g108780 | 60S ribosomal protein L7 (RPL7C) | TN115 |
| AT2G44640 | Medtr8g009290 | similar to PDE320 (PIGMENT DEFECTIVE 320) "Arabidopsis thaliana" (TAIR:AT3G06960.1); similar to unnamed protein product "Vitis vinifera" (GB:CAO70456.1) | TN115 |
| AT3G05060 | Medtr5g010260 | SAR DNA-binding protein putative | TN115 |
| AT3G05410 | Medtr1g083110 | similar to hypothetical protein OsI_004967 "Oryza sativa (indica cultivar-group)" (GB:EAY77120.1); similar to hypothetical protein OsJ_004568 "Oryza sativa (japonica cultivar-group)" (GB:EAZ14743.1) | TN115 |
| AT3G05560 | Medtr1g083430 Medtr1g088450 | 60S ribosomal protein L22-2 (RPL22B) | TN115 |
| AT3G07760 | Medtr5g097240 | similar to unnamed protein product "Vitis vinifera" (GB:CAO41197.1); contains InterPro domain Sterile alpha motif SAM (InterPro:IPR001660) | TN115 |
| AT3G10690 | Medtr1g031690 | DNA gyrase subunit A family protein | TN115 |
| AT3G12110 | Medtr2g008050 Medtr7g026230 | ACT11 (ACTIN-11); structural constituent of cytoskeleton | TN115 |
| AT3G12930 | Medtr2g069470 | similar to unknown "Populus trichocarpa" (GB:ABK94112.1); contains InterPro domain Iojap-related protein (InterPro:IPR004394) | TN115 |
| AT3G18680 | Medtr4g053800 Medtr8g093330 | aspartate/glutamate/uridylate kinase family protein | TN115 |
| AT3G22300 | Medtr1g005820 Medtr1g006120 | RPS10 (RIBOSOMAL PROTEIN S10); structural constituent of ribosome | TN115 |
| AT3G48730 | Medtr3g118070 | GSA2 (GLUTAMATE-1-SEMIALDEHYDE 21-AMINOMUTASE 2); glutamate-1-semialdehyde 21-aminomutase | TN115 |
| AT3G52150 | Medtr3g027140 | RNA recognition motif (RRM)-containing protein | TN115 |
| AT3G53480 | Medtr4g123850 | ATPDR9/PDR9 (PLEIOTROPIC DRUG RESISTANCE 9); ATPase coupled to transmembrane movement of substances | TN115 |
| AT3G53740 | Medtr1g100960 | 60S ribosomal protein L36 (RPL36B) | TN115 |
| AT3G54280 | Medtr4g035100 | ATP binding / DNA binding / helicase | TN115 |
| AT3G55280 | Medtr2g096340 Medtr4g063060 | 60S ribosomal protein L23A (RPL23aB) | TN115 |
| AT3G56070 | Medtr7g116340 | ROC2 (rotamase CyP 2); peptidyl-prolyl cis-trans isomerase | TN115 |
| AT3G56150 | Medtr1g023190 Medtr2g005610 Medtr7g116960 Medtr2g048870 Medtr6g045930 Medtr6g046050 | EIF3C (EUKARYOTIC TRANSLATION INITIATION FACTOR 3) | TN115 |
| AT4G00100 | Medtr4g102170 Medtr5g011910 | ATRPS13A (RIBOSOMAL PROTEIN S13A); structural constituent of ribosome | TN115 |
| AT4G18480 | Medtr2g015390 | CHLI1 (CHLORINA 42); magnesium chelatase | TN115 |
| AT4G22380 | Medtr4g114330 | ribosomal protein L7Ae/L30e/S12e/Gadd45 family protein | TN115 |
| AT4G26210 | Medtr5g062980 | mitochondrial ATP synthase g subunit family protein | TN115 |
| AT4G26370 | Medtr4g083370 | antitermination NusB domain-containing protein | TN115 |
| AT4G27720 | Medtr3g092030 | similar to unknown protein "Arabidopsis thaliana" (TAIR:AT1G64650.2) | TN115 |
| AT4G27760 | Medtr6g081020 | FEY (FOREVER YOUNG); oxidoreductase | TN115 |
| AT4G29510 | Medtr6g091770 | ATPRMT11/PRMT11 (ARABIDOPSIS ARGININE METHYLTRANSFERASE 11); protein-arginine N-methyltransferase | TN115 |
| AT4G31340 | Medtr3g109330 | myosin heavy chain-related | TN115 |
| AT4G32605 | Medtr5g093000 | transcription factor | TN115 |
| AT4G35260 | Medtr5g023740 Medtr8g074030 | IDH1 (ISOCITRATE DEHYDROGENASE 1); isocitrate dehydrogenase (NAD ) | TN115 |
| AT4G36420 | Medtr8g076780 | ribosomal protein L12 family protein | TN115 |
| AT4G37640 | Medtr4g096990 Medtr5g015590 | ACA2 (CALCIUM ATPASE 2); calmodulin binding | TN115 |
| AT5G01010 | Medtr8g005040 | similar to unnamed protein product "Vitis vinifera" (GB:CAO45344.1) | TN115 |
| AT5G04600 | Medtr1g108290 Medtr4g127330 | RNA recognition motif (RRM)-containing protein | TN115 |
| AT5G05010 | Medtr2g101180 | clathrin adaptor complexes medium subunit-related | TN115 |
| AT5G08160 | Medtr4g112900 | ATPK3 (Arabidopsis thaliana serine/threonine protein kinase 3); kinase | TN115 |
| AT5G08180 | Medtr4g112780 Medtr5g077600 | ribosomal protein L7Ae/L30e/S12e/Gadd45 family protein | TN115 |
| AT5G11420 | Medtr1g011800 Medtr2g103170 | similar to unknown "Ricinus communis" (GB:CAB02653.1); contains InterPro domain Galactose-binding like (InterPro:IPR008979) | TN115 |
| AT5G11950 | Medtr1g015830 | Encodes a protein of unknown function. It has been crystallized and shown to be structurally almost identical to the protein encoded by At2G37210. | TN115 |
| AT5G13300 | Medtr8g100100 | SFC (SCARFACE) | TN115 |
| AT5G13780 | Medtr3g034200 | GCN5-related N-acetyltransferase putative | TN115 |
| AT5G17020 | Medtr4g077660 Medtr8g013700 Medtr5g007790 | XPO1A (exportin 1A); protein transporter | TN115 |
| AT5G17710 | Medtr7g087250 | EMB1241 (EMBRYO DEFECTIVE 1241); adenyl-nucleotide exchange factor/ chaperone binding / protein binding / protein homodimerization | TN115 |
| AT5G18650 | Medtr2g020040 | zinc finger (C3HC4-type RING finger) family protein | TN115 |
| AT5G19750 | Medtr7g088590 | peroxisomal membrane 22 kDa family protein | TN115 |
| AT5G20180 | Medtr4g114610 | ribosomal protein L36 family protein | TN115 |
| AT5G20890 | Medtr7g067460 Medtr7g113470 Medtr7g067470 | chaperonin putative | TN115 |
| AT5G48630 | Medtr7g055650 | cyclin family protein | TN115 |
| AT5G50375 | Medtr3g089010 | CPI1 (CYCLOPROPYL ISOMERASE) | TN115 |
| AT5G51880 | Medtr1g101840 | oxidoreductase acting on paired donors with incorporation or reduction of molecular oxygen 2-oxoglutarate as one donor and incorporation of one atom each of oxygen into both donors | TN115 |
| AT5G53070 | Medtr5g038540 | ribosomal protein L9 family protein | TN115 |
| AT5G62000 | Medtr8g100050 | ARF2 (AUXIN RESPONSE FACTOR 2); transcription factor | TN115 |
| AT5G64813 | Medtr8g091980 | LIP1 (LIGHT INSENSITIVE PERIOD1); GTPase | TN115 |
| AT5G66750 | Medtr5g020000 | CHR01/DDM1 (DECREASED DNA METHYLATION 1); helicase | TN115 |
| AT5G67570 | Medtr4g097120 | EMB1408 (EMBRYO DEFECTIVE 1408) | TN115 |
